# The Impact of Internet Use on the Parameters of Attention in Adults

**DOI:** 10.1192/j.eurpsy.2024.332

**Published:** 2024-08-27

**Authors:** A. Mihai, M. Rebeca-Isabela

**Affiliations:** ^1^Psychiatry, University of Medicine, Pharmacy, Science and Technology; ^2^Mureș County Hospital, Targu-Mures , Romania

## Abstract

**Introduction:**

Internet use in the adult population is growing at alarming rates. The latest statistical data show an average internet usage time of 6 hours and 58 minutes (2023), an increase of 1% compared to 2021. Research studies on the influence of the excess use of internet on attention is in its prime years, and clear steps need to be made in an attempt to clarify current hypotheses and to find effective methods for prevention. Nowadays, one of the most powerful influences on attention is the use of the internet, which, more often than not, crosses the line of addiction.

**Objectives:**

The initial hypothesis is that in the event of exposure to a high number of stimuli, the ability to switch attention to a single task may only be possible at a superficial level. The aim of this study was to assess the impact that excess internet use has on the ability to maintain attention in the adult population. The present study aims to sketch a well-established structure and direction of research in the field of attention and its effects on human functioning.

**Methods:**

Using the DSM 5-TR diagnostic criteria for pathological Internet gaming disorder we enrolled 60 people who expressed their consent to participate in the study. We check psychiatric comorbidities using SCID II. As a method for evaluating changes in the level of attention, we used of the Stroop test. The results were analysed with the SPSS program (version 23).

**Results:**

The results showed a marked decrease in the ability to maintain attention, without increasing the number of stimuli. Although excessive Internet use leads to changes in attention parameters, research in this area is scarce and incomplete. Currently, most of the published studies focus on a causal relationship between the pathological use of the Internet and the appearance of attention deficit/hyperkinetic disorder, especially in children and adolescents. Although the results are promising, we cannot neglect the multitude of additional consequences of excess Internet use, which these studies targeting a single pathology overlook. Moreover, using the Internet involves exposure to an ever-increasing number of stimuli, which is why switching attention and maintaining it is currently an insufficiently researched parameter. Regarding the impact of Internet use on individual functioning, there is a relatively modest number of studies in the literature that outline a correlation between excess Internet use and various psychiatric comorbidities.

**Conclusions:**

The impact of the research on the general population could be an increased awareness of negative effects and the development of prevention programs.

**Disclosure of Interest:**

None Declared

